# Identification and validation of efferocytosis-related biomarkers for the diagnosis of metabolic dysfunction-associated steatohepatitis based on bioinformatics analysis and machine learning

**DOI:** 10.3389/fimmu.2024.1460431

**Published:** 2024-10-21

**Authors:** Chenghui Cao, Wenwu Liu, Xin Guo, Shuwei Weng, Yang Chen, Yonghong Luo, Shuai Wang, Botao Zhu, Yuxuan Liu, Daoquan Peng

**Affiliations:** ^1^ Department of Cardiology, Research Institute of Blood Lipids and Atherosclerosis, The Second Xiangya Hospital, Central South University, Changsha, Hunan, China; ^2^ Xiangya School of Medicine, Central South University, Changsha, China

**Keywords:** metabolic dysfunction-associated steatohepatitis, efferocytosis, bioinformatic analysis, machine learning, TREM2, TIMD4

## Abstract

**Background:**

Metabolic dysfunction-associated steatohepatitis (MASH) is a highly prevalent liver disease globally, with a significant risk of progressing to cirrhosis and even liver cancer. Efferocytosis, a process implicated in a broad spectrum of chronic inflammatory disorders, has been reported to be associated with the pathogenesis of MASH; however, its precise role remains obscure. Thus, we aimed to identify and validate efferocytosis linked signatures for detection of MASH.

**Methods:**

We retrieved gene expression patterns of MASH from the GEO database and then focused on assessing the differential expression of efferocytosis-related genes (EFRGs) between MASH and control groups. This analysis was followed by a series of in-depth investigations, including protein–protein interaction (PPI), correlation analysis, and functional enrichment analysis, to uncover the molecular interactions and pathways at play. To screen for biomarkers for diagnosis, we applied machine learning algorithm to identify hub genes and constructed a clinical predictive model. Additionally, we conducted immune infiltration and single-cell transcriptome analyses in both MASH and control samples, providing insights into the immune cell landscape and cellular heterogeneity in these conditions.

**Results:**

This research pinpointed 39 genes exhibiting a robust correlation with efferocytosis in MASH. Among these, five potential diagnostic biomarkers—*TREM2, TIMD4, STAB1, C1QC, and DYNLT1*—were screened using two distinct machine learning models. Subsequent external validation and animal experimentation validated the upregulation of *TREM2* and downregulation of *TIMD4* in MASH samples. Notably, both *TREM2* and *TIMD4* demonstrated area under the curve (AUC) values exceeding 0.9, underscoring their significant potential in facilitating the diagnosis of MASH.

**Conclusion:**

Our study comprehensively elucidated the relationship between MASH and efferocytosis, constructing a favorable diagnostic model. Furthermore, we identified potential therapeutic targets for MASH treatment and offered novel insights into unraveling the underlying mechanisms of this disease.

## Introduction

Metabolic dysfunction-associated fatty liver disease (MASLD), formerly termed as non-alcoholic fatty liver disease (NAFLD), is the most prevalent chronic liver ailment, with a global incidence rate of approximately 30% (1, 2). MASLD encompasses two main subtypes: simple fat infiltration, commonly known as metabolic dysfunction-associated steatotic liver (MASL), and metabolic dysfunction-associated steatohepatitis (MASH) (3). The latter subtype, MASH, replaces the preceding term non-alcoholic steatohepatitis (NASH), representing an inflammatory variant of MASLD. The prominent pathological features of MASH are excessive accumulation of fat in hepatocytes, accompanied with steatosis, ballooning, and lobular inflammation, with or without perisinusoidal fibrosis (4, 5). Although simple steatosis poses a relatively low risk of developing cirrhosis, its transition to MASH significantly elevates this risk (6, 7). The clinical symptoms of MASH patients are often subtle, and liver biopsy serves as the primary diagnostic criterion. However, liver aspiration being an invasive procedure, requiring expert medical personnel, renders it unsuitable for widespread screening. Consequently, the identification of novel and effective MASH biomarkers holds paramount importance for the prompt diagnosis and treatment of this condition.

Efferocytosis, representing the ultimate mechanism for eliminating dead cells, is pivotal in maintaining bodily homeostasis under physiological circumstances and fostering tissue restoration in pathological states (8). The phagocytes involved in this process can be broadly classified into two groups: professional phagocytes, such as macrophages and dendritic cells (9), and non-professional phagocytes, including vascular smooth muscle cells (VSMCs) and endothelial cells (ECs), etc., which exhibit efferocytic capabilities under specific conditions (10, 11). Current research categorizes efferocytosis into three distinct phases: ‘find me’, ‘eat me’ and ‘digest me’ (12). In the initial ‘find me’ phase, apoptotic cells release signaling molecules like ATP, lysophosphatidylcholine (LPC), and sphingosine 1-phosphate (S1P) to notify and attract phagocytes (13–16). Subsequently, in the ‘eat me’ phase, phagocytes distinguish living cells from dying cells by recognizing phosphatidylserine (PS) exposed on the surface of apoptotic cells (17, 18). The PS-recognizing receptors on phagocyte membranes can be grouped into two categories. The first category consists of receptors that directly bind to PS, such as the brain-specific angiogenesis inhibitor 1 (BAI1) (19), Stabilin1/2 (20, 21), and T cell immunoglobulin mucin receptors TIM1/4 (22, 23). The second category comprises receptors that require bridge molecules to indirectly recognize PS, including growth arrest-specific protein 6 (GAS6)/protein S for the TAM receptor family (24) and milk fat globule-EGF factor 8 (MFG-E8) for the αvβ3/5 integrins (25, 26). Finally, in the ‘digest me’ phase, the phagocytes degrade the internalized apoptotic cell and then secrete cytokines like IL-10 and transforming growth factor-β (TGFβ) to exert anti-inflammatory effects and promote damaged tissue repair (27, 28).

Over the past decades, research has illuminated the significance of efferocytosis in multiple diseases, including cardiovascular diseases, metabolic disorders, and cancer, etc. The malfunction of efferocytosis often exacerbates disease progression, whereas enhancing efferocytosis can make improvements. However, our comprehension of efferocytosis’s role in MASH remains limited. Further exploration of efferocytosis in MASH could potentially uncover novel diagnostic biomarkers and therapeutic strategies.

In this study, differential gene analysis of liver transcriptome data from 16 MASH patients and 14 healthy controls in the GSE126848 dataset of the GEO database revealed 39 genes related to MASH and efferocytosis. Among these, five hub genes—*TREM2, TIMD4, STAB1, C1QC, and DYNLT1*—were identified using two robust machine learning methods. Subsequent immune infiltration and single-cell transcriptome analyses confirmed the altered expression level of these hub genes in immune cells. To strengthen our findings, validation was performed using external datasets (GSE246221) as well as animal experiment. Our research is anticipated to offer novel targets for the diagnosis and therapeutic intervention of MASH.

## Methods and materials

### Data preparation

By searching the Gene Expression Omnibus (GEO) database (https://www.ncbi.nlm.nih.gov/geo/), we identified GSE126848 as our primary test dataset, featuring liver transcriptome profiles of 14 healthy controls and 16 MASH patients (29). For validation, we selected GSE246221, which comprises liver microarray data from 4 healthy controls and 28 MASLD patients (30). Based on the MASLD activity score, 22 samples were diagnosed with MASH (31). Therefore, subsequent gene expression validation and diagnostic capability verification will utilize 4 healthy controls and 22 MASH samples from this dataset. Additionally, we incorporated GSE128334, a single-cell RNA sequencing (scRNA-seq) dataset from MASH mice model, to assess hub gene expression specifically in immune cells (32). To gather efferocytosis related genes (EFRGs), we queried the Gene Cards database (https://www.genecards.org/) and the Kyoto Encyclopedia of Genes and Genomes (KEGG) database (https://www.genome.jp/kegg/), focusing on the keyword “efferocytosis”. This thorough search yielded a total of 272 EFRGs, as detailed in **Supplementary Table S1**.

### Differentially expressed genes analysis

First, the raw counts of GSE126848 were normalized using the Trimmed Mean of M-values (TMM) method from edgeR package version 4.0.16 within the R software version 4.3.3. Subsequently, the data distribution was examined, and a PCA cluster plot was generated to visualize the patterns. Second, to identify differentially expressed genes (DEGs) between MASH patients and healthy controls in the training dataset, we employed the DESeq2 package version 1.42.1. Twenty percent of the genes with very low expression levels were filtered out, and subsequently, those with p-adj < 0.05 and |log2 FC| ≥ 1 were identified as DEGs. To visualize the DEGs, volcano plots were generated. Following the intersection of DEGs with the EFRGs, the differentially expressed EFRGs in MASH was determined. Finally, the Venn Diagram package version 1.7.3 and ggplot2 package version 3.5.0 were utilized to visualize the results in a Venn diagram and heat map.

### Functional enrichment analysis

After identifying differentially expressed EFRGs, we conducted a series of enrichment analyses utilizing the clusterProfiler package, version 4.10.1. This process encompassed Gene Ontology (GO) enrichment analysis, specifically targeting Biological Process (BP) and Molecular Function (MF) categories. Additionally, we performed Kyoto Encyclopedia of Genes and Genomes (KEGG) pathway enrichment analysis and Disease Ontology (DO) enrichment analysis. Finally, we filtered and visualized the top-ranking results with a p-value < 0.05.

### Machine learning

As artificial intelligence technology advances, it is increasingly applied to screening novel diagnostic biomarker for disease. In this research, we employed two machine learning methods, Least Absolute Shrinkage and Selection Operator (LASSO) and Support Vector Machine Recursive Feature Elimination (SVM-RFE), to further refine the selection of hub genes for the diagnosis of MASH (33, 34). Based on the previously identified 39 EFRGs related to MASH, 7 and 13 hub genes were filtered out by LASSO and SVM-RFE respectively, ultimately yielding 5 hub genes through the intersection of the two lists. For LAASO regression, the model was specified with the binomial parameter. The optimal lambda value was chosen based on lambda.min, and the model was validated using 10-fold cross-validation. For SVM-RFE, five-fold cross-validation was utilized for model evaluation. For more detailed information on the machine learning approach, please refer to the GitHub repository: https://github.com/chenghui3595/MASH-Efferocytosis-ML.

### Construction of ROC and nomogram model

To assess the diagnostic value of hub genes, we constructed the Receiver Operating Characteristic (ROC) curves, which were conducted by using the “pROC” package version 1.18.5. ROC curves demonstrated the Area Under the Curve (AUC), along with specificity and sensitivity. To reinforce the validity of marker genes, an external dataset, GSE246221, was employed to verify the diagnostic capability. Furthermore, a nomogram model was devised utilizing the “rms” package version 6.8-0 for predicting the onset of MASH. In the nomogram, each hub gene is assigned a distinct score, and the “total points” are derived by summing the scores of all contributing predictors.

### Individual gene GSEA

The correlation between the five hub genes and other genes was calculated individually, and a correlation gene set was identified based on the strength of these correlations. Subsequently, a gene set enrichment analysis (GSEA) was conducted utilizing the clusterProfiler package v4.10.1. To gain further insights, we compared the KEGG pathways between MASH patients and healthy controls, ultimately visualizing the top five upregulated and downregulated pathways, respectively.

### Assessment of the immune infiltration

CIBERSORT algorithm can assess the relative abundance of 22 distinct immune cell types in both MASH and healthy control groups, solely based on gene expression profiles (35). This process was carried out by “CIBERSORT” package version 0.1.0. The proportion of the immune cell infiltration between MASH and the healthy control groups was visualized by boxplot. The correlation between the hub genes and significant infiltrated immune cell was investigated using Spearman’s correlation analysis.

### Analysis of single-cell transcriptome data

The scRNA-seq dataset GSE128334 of mouse liver was downloaded from the GEO database, encompassing two MASH samples and two control samples (32). We initially created Seurat objects by systematically importing the single-cell data. Following this, quality control is executed, primarily focusing on the count of genes expressed per cell and the proportion of mitochondrial genes. Subsequently, we integrated the four Seurat objects and remove the batch effect at the same time. Then, this prepared data utilized PCA to reduce the feature dimensions and t-Distributed Stochastic Neighbor Embedding (t-SNE) to identify distinct cellular clusters. Finally, to characterize these clusters, we identified marker genes and annotated each cluster. The “Seurat” package version 5.1.0 was employed throughout this process.

### PPI and TF-miRNA-mRNA regulatory network construction

Protein–protein interaction (PPI) network of 39 differentially expressed EFRGs was generated using the STRING database (https://cn.string-db.org/). For this PPI analysis, a medium confidence threshold of 0.25 was set. Additionally, a regulatory network involving Transcription Factor (TF), microRNA (miRNA), and mRNA was constructed on the NetworkAnalyst platform (https://www.networkanalyst.ca/NetworkAnalyst/). In this research, Transcription factors were derived from the ChEA database, and miRNA-gene interaction data were collected from the TarBase v8.0 database. Both results were visualized by Cytoscape version 3.10.2.

### MASH mice model construction

In this study, we employed male C57BL/6J mice aged 6 to 8 weeks and induced the MASH model by subjecting them to a 28-week regimen of a high-fat diet (HFD) coupled with intraperitoneal injections of streptozotocin (STZ). Specifically, the mice received STZ injections at a dose of 40 mg/kg for five consecutive days. The HFD, sourced from Research Diets, Inc, under the product code D12450B. The caloric content of the diet was designed as follows: 20% from protein, 20% from carbohydrates, and 60% from fats.

### RNA extraction and quantitative real-time PCR

Total RNA was extracted from MASH and control mouse tissues utilizing the AG RNAex Pro Reagent (AG21101, China). This RNA was then converted into cDNA using the Evo M-MLV Reverse Transcription Kit (AG11706, China). For quantitative analysis of gene expression, quantitative real-time PCR (qRT-PCR) was conducted on the CFX Connect system (Bio-Rad, USA) with SYBR^®^ Green Supermix (Bio-Rad, USA). The expression levels of hub genes were quantified using the 2^-ΔΔCT method, with *Gapdh* serving as a stable internal control for normalization. The specific primers employed in these qRT-PCR assays are detailed in **Supplementary Table S2**.

### Biochemical detection of serum

During the last week of the study, blood samples were collected from the tail vein of mice after a 4-hour fast to quantify serum metabolites. These samples were centrifuged at 3000g for 15 minutes at 4°C to isolate the serum, which was subsequently stored at -80°C. The levels of serum ALT and AST were measured using commercial assay kits (Elabscience, China) in accordance with the manufacturers’ protocols.

### Histological analysis

For histological analysis, mouse liver tissue was first fixed in 4% paraformaldehyde (P0099, Beyotime, China) and then embedded in paraffin. The tissues were subsequently sectioned, and histological changes were assessed using hematoxylin and eosin (H&E) staining (Solarbio, China) as well as Oil Red O staining (C1057S, Beyotime, China).

### Glucose tolerance test and insulin tolerance test

The oral glucose tolerance test (OGTT) and insulin tolerance test (ITT) were conducted separately. For the OGTT, mice were fasted overnight before receiving a D-glucose solution at a dosage of 1 g/kg body weight via oral gavage. Blood samples were collected from the tail vein at 0, 15, 30, 60, 90, and 120 minutes post-gavage to assess glucose clearance. For the ITT, mice were fasted for 6 hours and then given an intraperitoneal injection of insulin at a dosage of 0.75 U/kg. Blood samples were collected at 0, 15, 30, 60, 90, and 120 minutes post-injection to evaluate insulin sensitivity. Blood glucose levels were promptly measured using a glucometer (ACCU-CHEK Guide Me, China).

### Western blotting

Liver tissues from control and MASH model mice were finely minced and processed for protein extraction using the RIPA lysis buffer. Polyacrylamide gels (10%) were prepared with the Omni-Easy™ One-step Color PAGE Gel Rapid Preparation Kit (Cat No: PG210-214). The 10-180 kDa Prestained Protein Marker from Thermo (Cat No: 26616) was used for molecular weight estimation. Electrophoresis and membrane transfer were performed using the BIO-RAD PowerPac Basic Power Supply. Blocking was carried out with Beyotime Quick Block™ Western (Cat No: P0252). Primary antibodies were diluted as follows: β-tubulin (1:5000, Proteintech, Cat No: 10094-1-AP), TREM2 (1:1000, Abcam, Cat No: ab305103), and TIMD4 (1:1000, Affinity Biosciences, Cat No: DF13636). Grayscale values of all bands were quantified using ImageJ, and relative protein expression levels were normalized to β-tubulin. Statistical analysis and graphical representation were performed using GraphPad Prism 9.

### Immunohistochemistry

For immunohistochemistry (IHC), paraffin-embedded sections were dewaxed in xylene, rehydrated through an alcohol gradient, and subjected to antigen retrieval using EDTA solution in a microwave. To block nonspecific binding, the sections were incubated with normal goat serum for 90 minutes. Primary antibodies against TREM2 (Abcam, ab305103, 1:200) and TIMD4 (Affinity Biosciences, DF13636, 1:200) were applied and incubated overnight at 4°C. The next day, sections were re-warmed, treated with 3% hydrogen peroxide for 8 minutes, and rinsed with PBS. After incubation with secondary antibodies (1:200) for 1 hour at room temperature, the sections were stained using diaminobenzidine (DAB, Sigma, USA) and counterstained with hematoxylin. The stained slides were visualized and captured using a 3D HISTECH digital slide scanner.

## Results

### Identification of differentially expressed EFRGs

**Figure 1:**
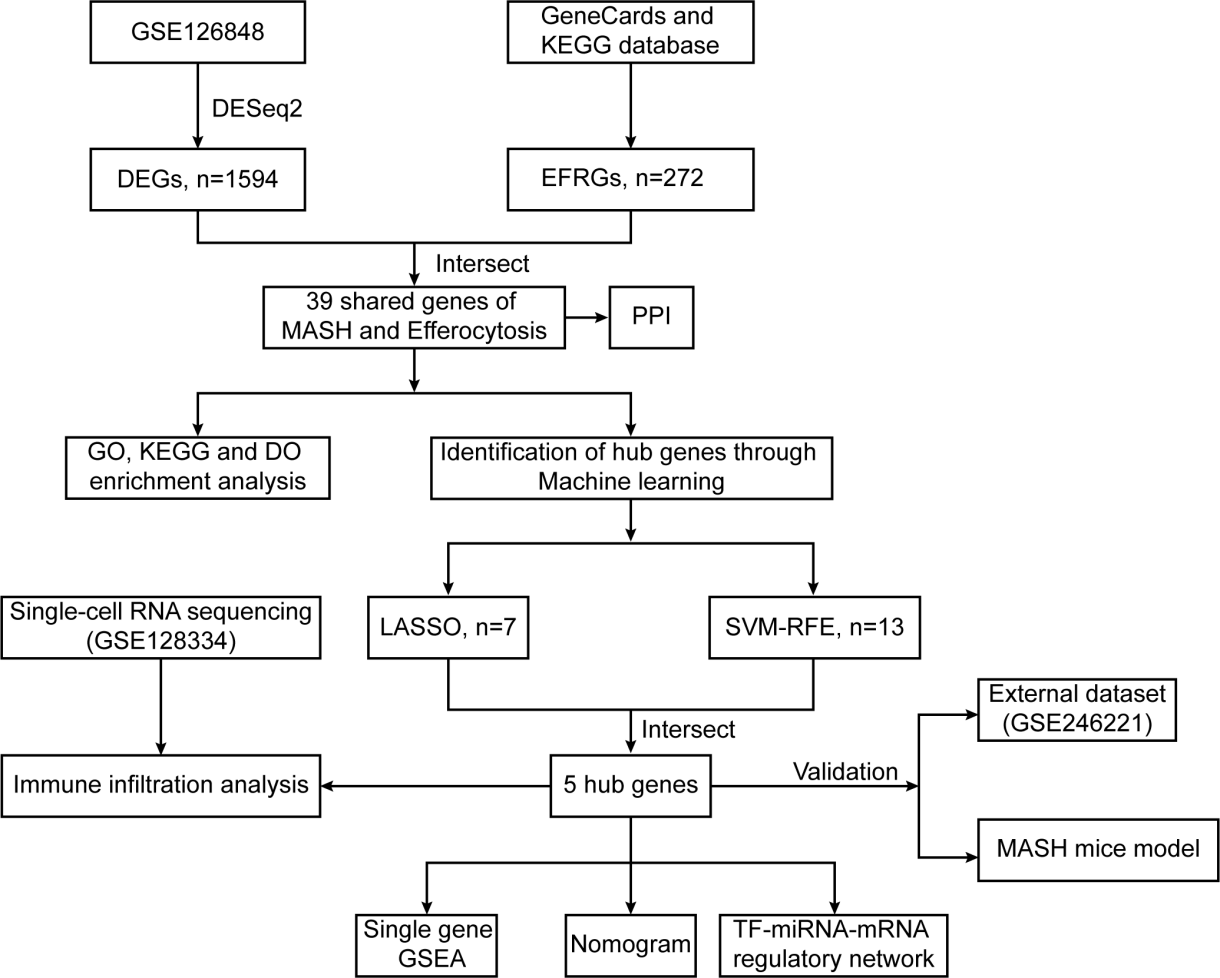
Flowchart illustrating the present study design. 5 hub genes (*TREM2, TIMD4, STAB1, C1QC and DYNLT1*) were identified by two machine learning algorithms.

**Figure 2:**
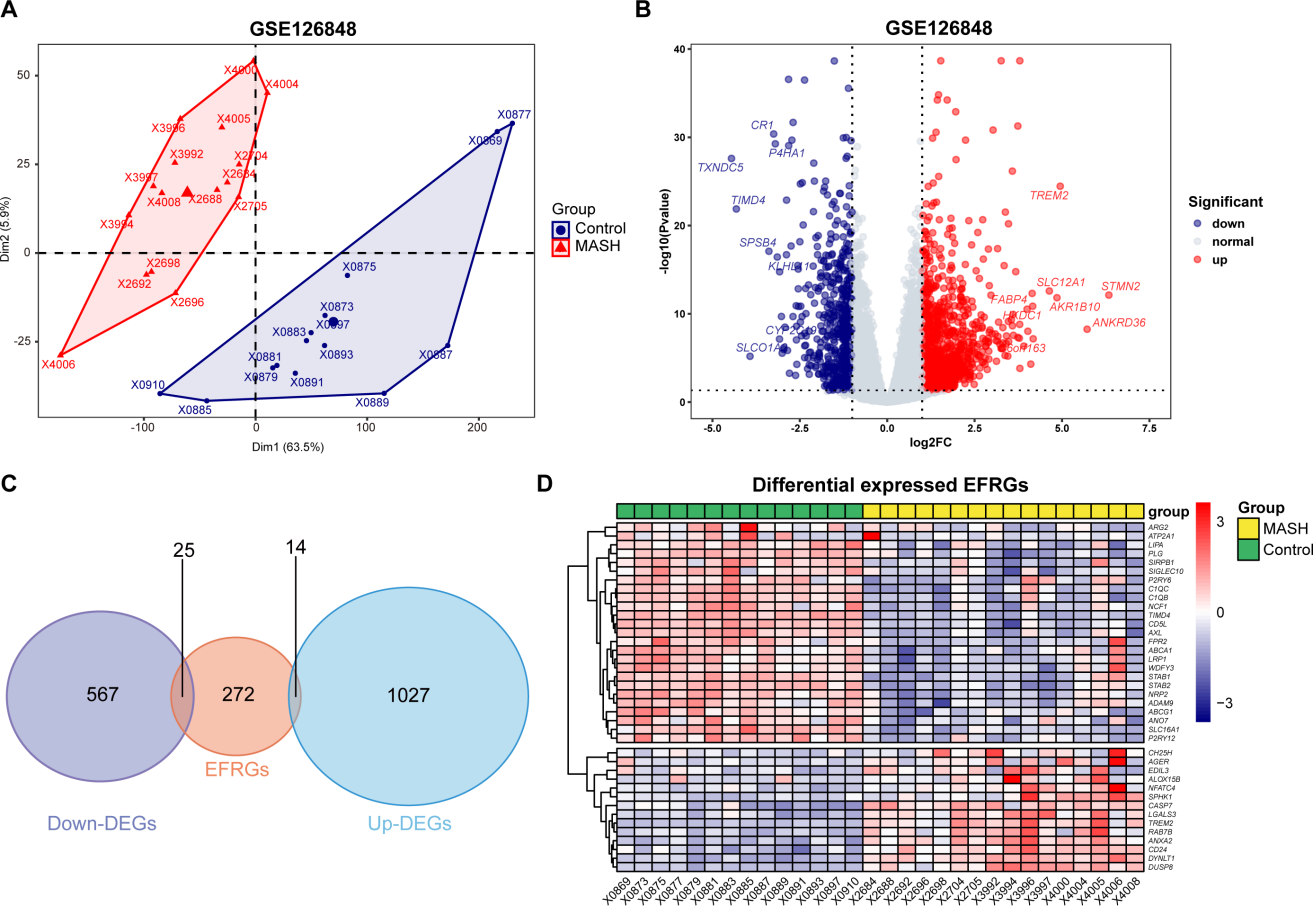
Identification of differentially expressed EFRGs. **(A)** PCA showed significant differences between the MASH and control liver samples. **(B)** Volcano plot showed the DEGs between MASH and control groups, and the top eight genes with the most significant fold changes was pointed. **(C)** Venn diagram showed the intersection of genes between DEGs and EFRGs. **(D)** Heatmaps showed the 39 intersected EFRGs. DEGs, differential expression genes; EFRGs, efferocytosis-related genes.

The data analysis process of this study is outlined in **Figure 1**. As **Figure 2A** demonstrates, a principal component analysis (PCA) revealed significant differences between the MASH and control groups. Utilizing the DESeq2 package, we identified a total of 1594 DEGs, with 1027 genes exhibiting upregulation and 567 genes showing downregulation. The findings of this analysis are visually presented in a volcano plot, highlighting the top eight genes with the most significant fold changes (**Figure 2B**). Subsequently, we intersected the list of DEGs with EFRGs and identified 39 differentially expressed EFRGs (**Figure 2C**). Finally, we created heatmaps to demonstrate their expression level in MASH patients and healthy controls (**Figure 2D**), which shows oversharp distinction.

### Functional enrichment analysis of 39 differentially expressed EFRGs

**Figure 3:**
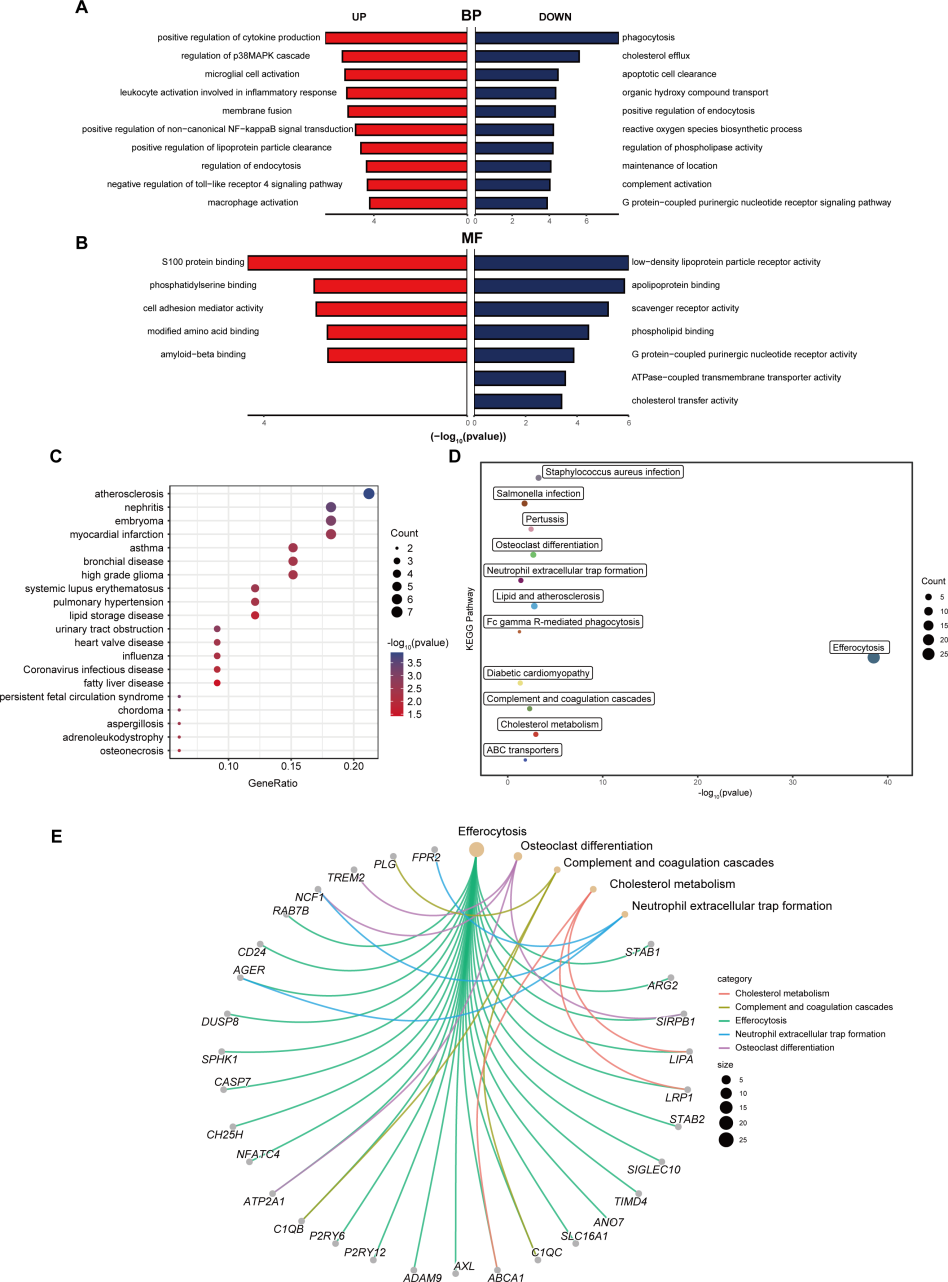
Functional enrichment analysis of differentially expressed EFRGs. **(A)** Butterfly diagram of the GO enrichment analysis of 39 up- and down-regulated EFRGs, Biological process (BP). **(B)** Butterfly diagram of the GO enrichment analysis of 39 up- and down-regulated EFRGs, Molecular function (MF). **(C)** Bubble diagrams of the DO enrichment analysis of 39 differentially expressed EFRGs. **(D)** The KEGG enrichment analysis plot displays the signaling pathways most closely related to the 39 intersecting EFRGs. **(E)** The KEGG enrichment analysis circular plot depicts a network of gene-pathway relationships.

To gain a profound comprehension of the biological implications of the 39 differentially expressed EFRGs, we conducted a suite of functional enrichment analyses leveraging Gene Ontology (GO), Disease Ontology (DO), and Kyoto Encyclopedia of Genes and Genomes (KEGG). The GO enrichment analysis revealed that, within the biological process (BP) categories, phagocytosis, cholesterol efflux, and apoptotic cell clearance pathways were downregulated, whereas the pathway related to positive regulation of cytokine production was upregulated (**Figure 3A**). Among the molecular function (MF) categories, activities such as low-density lipoprotein particle receptor binding, apolipoprotein binding, and scavenger receptor activity were downregulated, whereas S100 protein binding was upregulated (**Figure 3B**). The DO enrichment analysis highlighted a significant enrichment in atherosclerosis and nephritis (**Figure 3C**). Furthermore, the KEGG pathway enrichment analysis revealed a significant enrichment in efferocytosis, complement and coagulation cascades, neutrophil extracellular trap formation, osteoclast differentiation and cholesterol metabolism pathways (**Figures 3D, E**).

### Identification of hub genes using machine learning

**Figure 4:**
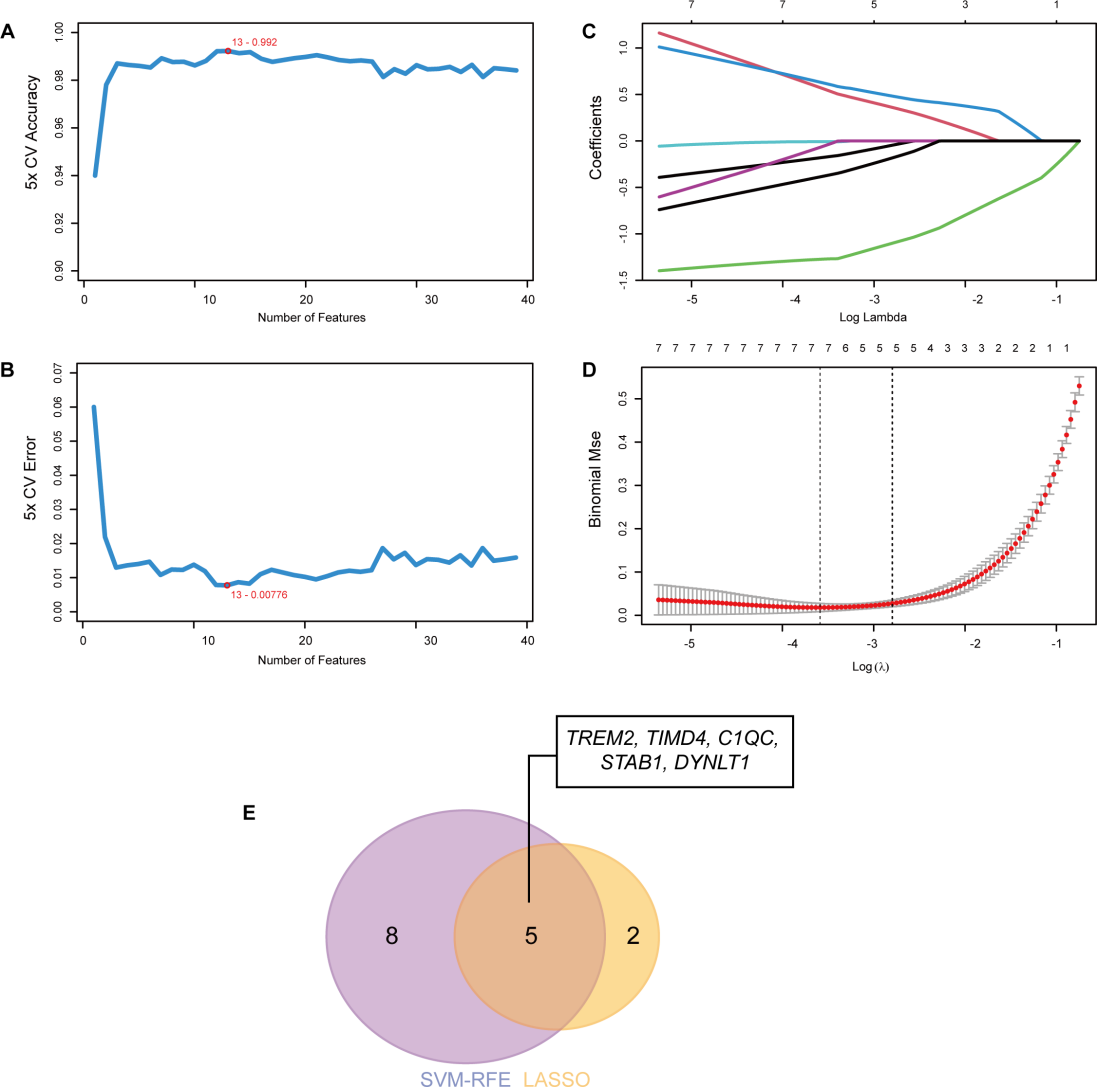
Identification of hub genes using machine learning. **(A)** The accuracy and **(B)** the error of the feature selection for the SVM-RFE algorithm. 13 genes were pinpointed. **(C, D)** 7 genes were identified through the LASSO regression algorithm. **(E)** The Venn diagram showed the overlap of hub genes between SVM-RFE and LASSO algorithms.

To further refine the selection of diagnostic genes capable of distinguishing MASH patients from healthy controls, we employed two machine learning algorithms: LASSO regression and SVM-RFE, based on the previously identified 39 EFRGs. The SVM-RFE algorithm identified 13 candidate genes (**Figures 4A, B**), while the LASSO regression algorithm narrowed down the list to an additional set of 7 genes (**Figures 4C, D**). By intersecting the results of both algorithms, we identified a set of 5 shared biomarkers: *TREM2, TIMD4, C1QC, STAB1*, and *DYNLT1* (**Figure 4E**). These genes represent promising diagnostic targets for further investigation.

### GSEA of hub genes

**Figure 5:**
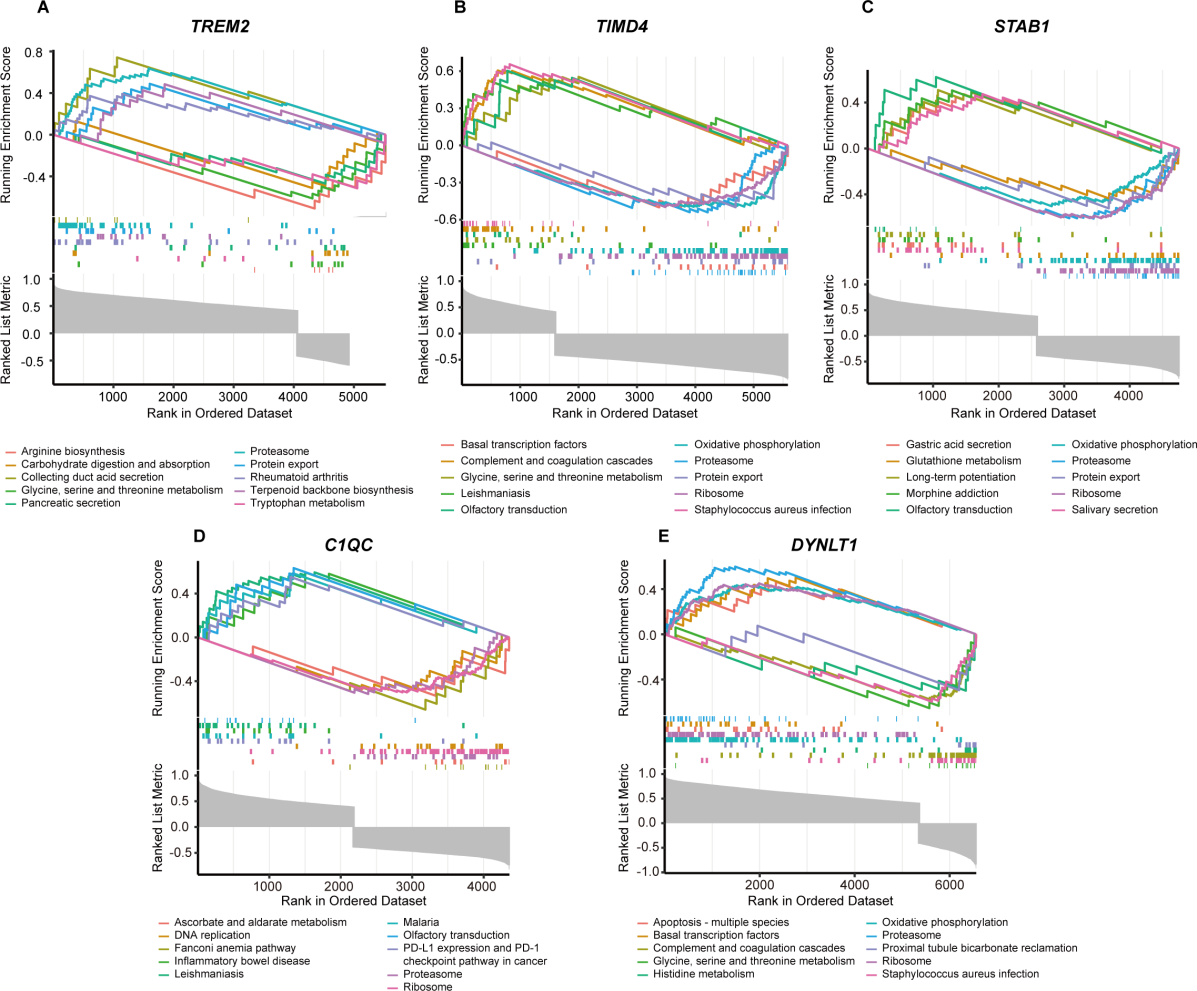
GSEA analysis of five marker genes. The KEGG pathway enrichment analysis of **(A)** *TREM2*, **(B)** *TIMD4*, **(C)** *STAB1*, **(D)** *C1QC* and **(E)** *DYNLT1* were conducted by GSEA enrichment method, and the five pathways with the highest and lowest enrichment scores are visualized according to the arrangement of enrichment scores.

Utilizing the KEGG pathways as a reference, we conducted individual gene GSEA to decipher the dominant signaling pathways relevant to our hub genes. Our GSEA of KEGG pathways revealed that low expression levels of the five key genes are implicated in pathways such as Oxidative Phosphorylation, Proteasome, Protein Export, and Ribosome. Conversely, high expression of these genes is associated with Amino Acid Metabolism (**Figures 5A–E**). Furthermore, we observed that *TIMD4* and *DYNLT1* exhibit connections with the Complement and Coagulation Cascades pathway (**Figures 5B, E**), while *TIMD4*, *STAB1*, and *C1QC* are linked to the Olfactory Transduction pathway (**Figures 5B–D**).

### Construction of PPI network and TF-miRNA-mRNA regulatory network

**Figure 6:**
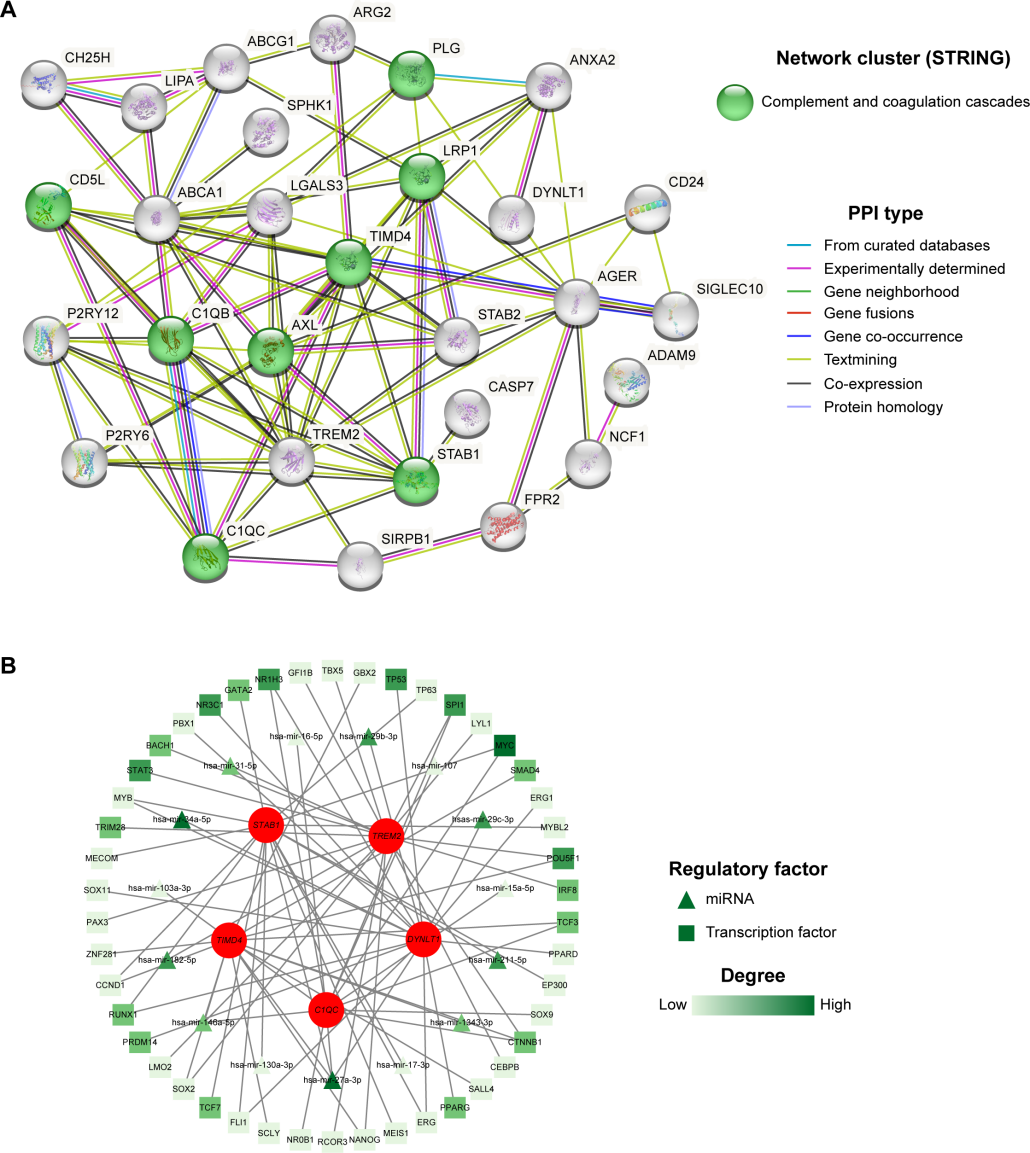
PPI and TF-miRNA-mRNA regulatory network. **(A)** 39 intersected genes of MASH and efferocytosis were explored within the PPI network. **(B)** TF-miRNA-mRNA regulatory network of the five hub genes.

**Figure 6A** demonstrated the PPI network of 39 differentially expressed EFRGs. From the results of the PPI analysis, we observed that *C1QC*, *TIMD4*, *TREM2*, and *STAB1* are closely associated with other differentially expressed EFRGs, further confirming their potential as core genes influencing MASH. Additionally, the STRING database revealed that these 39 differentially expressed genes are significantly enriched in the complement and coagulation cascades pathway, which aligned with our previous functional enrichment results. This suggested that EFRGs may regulate MASH by affecting this pathway. Utilizing the Network Analyst platform, we further predicted potential TFs and miRNAs for five hub genes, leveraging the ChEA database and TarBase v8.0 respectively. **Figure 6B** depicted potential transcription factors and miRNAs that modulate the expression of these hub genes. By ranking the degrees of connectivity, we identified a transcription factor, MYC, and two miRNAs, miR-34a-5p and miR-27a-3p, which play crucial roles in regulating the expression of the core genes. Overall, the roles of these regulatory factors and core genes in MASH warrant further investigation.

### Immune infiltration analysis and scRNA-seq dataset validation

**Figure 7:**
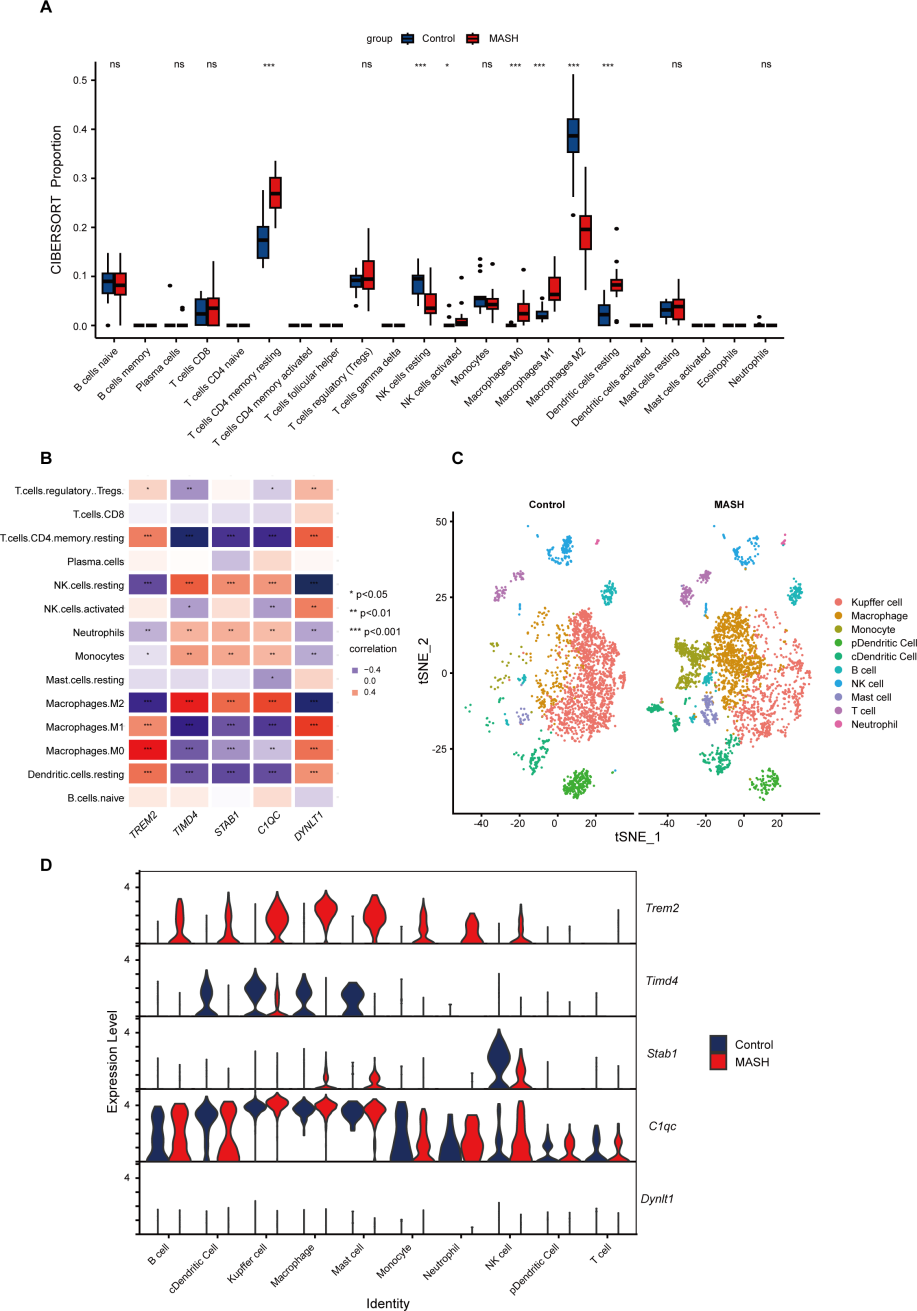
Immune Infiltration and scRNA-Seq analysis between MASH and control groups. **(A)** Boxplots showed immune infiltration differences between MASH and control samples. 7/22 immune cells significantly differ in MASH and controls. **(B)** The relationship between varied immune cell infiltration and five marker genes is depicted, with red indicating positive correlation and blue indicating negative correlation. **(C)** t-SNE plot showed the 10 identified cell clusters of the scRNA-Seq dataset GSE128334. **(D)** Violin plot demonstrated the five hub genes expression level in different cell clusters between MASH and control groups. ns indicates not significant, * indicates P<0.05, ** indicates P<0.01, *** indicates P<0.001.

MASH is an inflammatory disease marked by the penetration of immune cells into plaques and hepatic lobule. Efferocytosis has also been reported to play a regulatory role in the modulation of inflammation. To elucidate whether efferocytosis contributes to MASH progression by modulating immune cell infiltration, we conducted a CIBERSORT analysis. This algorithm allowed us to assess the disparities in the immune microenvironment between MASH patients and healthy controls. As depicted in **Figure 7A**, the proportions of 22 distinct immune cell types were analyzed, revealing significant differences in the expression of seven immune cell subsets. Specifically, we observed a higher abundance of resting CD4 memory T cells, activated NK cells, resting Dendritic cells, Macrophages M0, and Macrophages M1 in MASH patients. Conversely, resting NK cells and M2 macrophages were less prevalent. Subsequently, we conducted a correlation analysis of hub genes based on the infiltrating immune cell types (**Figure 7B**). This analysis revealed a positive correlation between *TREM2* and *DYNLT1* in M0 macrophages, whereas a negative correlation was observed in M2 macrophages. Beyond that, *TIMD4, STAB1*, and *C1QC* exhibited a positive correlation in M2 macrophages and a negative correlation in M1 macrophages.

To gain further insights into the distribution of immune cells in the livers of MASH and control, we performed a scRNA-Seq analysis on the livers of MASH mice model. As illustrated in **Figure 7C**, the proportion of Kupffer cells, representing M2 macrophages, was reduced in the livers of MASH mice, while there was an increased infiltration of the mononuclear macrophage derived from the blood. These findings align with our CIBERSORT immune infiltration analysis. **Figure 7D** underscored the elevated expression levels of *TREM2* in various immune cells, while the expression of *TIMD4* is reduced.

### Validation of hub genes diagnostic value and expression

**Figure 8:**
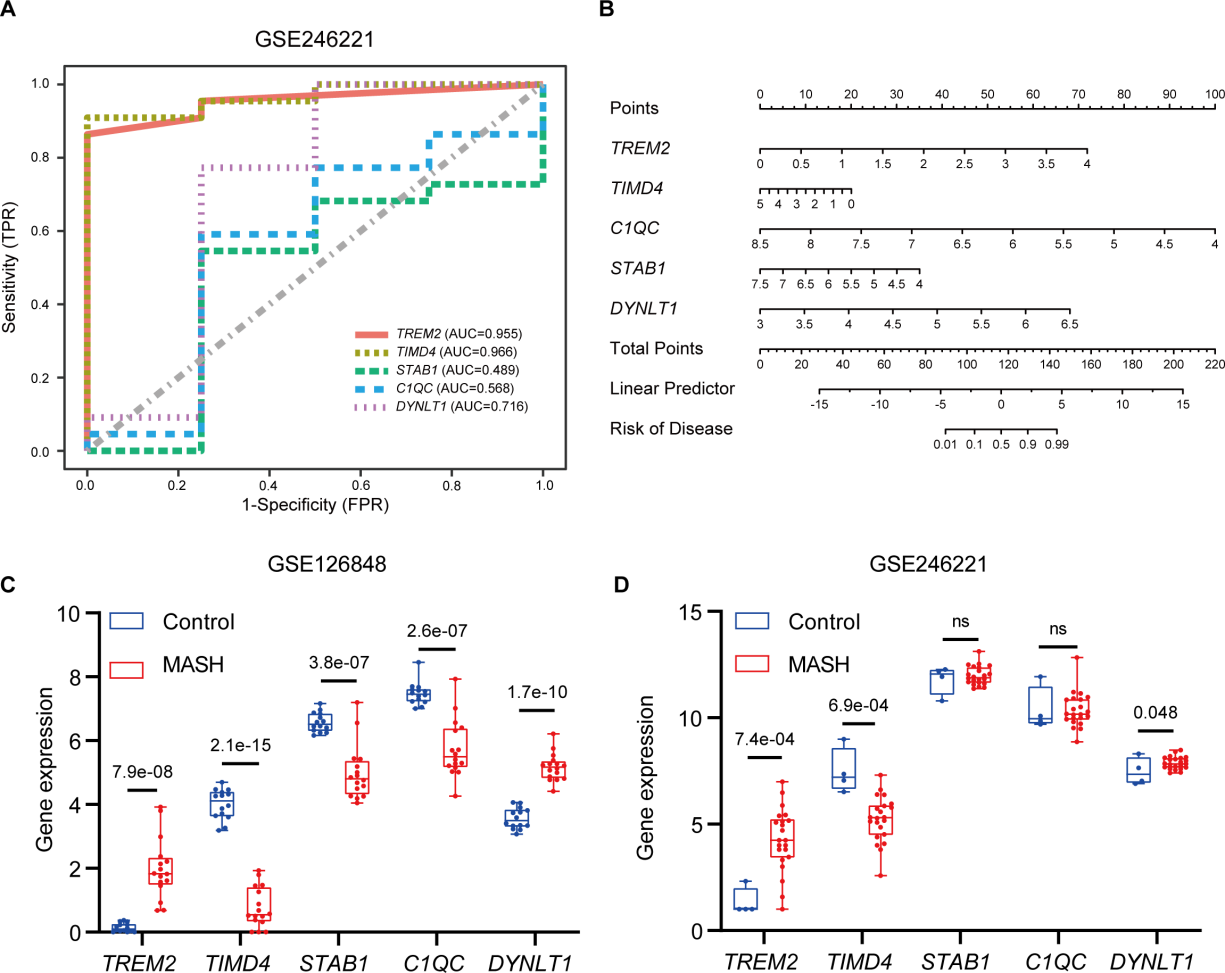
Validation of marker gene expression. **(A)** The ROC results of five marker genes in GSE246221. The AUC value of *TREM2*, *TIMD4, STAB1, C1QC* and *DYNLT1* was 0.955, 0.966, 0.489, 0.568, 0,716 respectively. **(B)** Nomogram for the diagnosis of MASH based on the hub genes. **(C)** Boxplots indicating the five differentially expressed EFRGs between MASH and control samples in GSE126848. **(D)** Boxplots indicating the five differentially expressed EFRGs between MASH and control samples in GSE246221.

The diagnostic value of hub genes in identifying MASH was assessed using the GSE246221 dataset, with ROC curve analysis. Notably, *TREM2* (AUC: 0.955) and *TIMD4* (AUC: 0.966) emerged as potent biomarkers, each demonstrating significant diagnostic value for MASH (**Figure 8A**). To enhance the predictive prowess of these hub genes, we then constructed a comprehensive nomogram model tailored specifically for MASH patients. This model integrates *TREM2, TIMD4, STAB1, C1QC*, and *DYNLT1*, assigning a unique score to each biomarker (**Figure 8B**). By summing these scores, the model enables the prediction of MASH risk, with *TREM2, C1QC*, and *DYNLT1* exhibiting particularly satisfactory diagnostic performance. In a complementary analysis utilizing the GSE126848 dataset, we observed *TIMD4*, *STAB1*, and *C1QC* expression level were downregulated in MASH patients, whereas *TREM2* and *DYNLT1* exhibited upregulation (**Figure 8C**). These findings were subsequently corroborated in the GSE246221 dataset (**Figure 8D**).

However, it is important to acknowledge certain limitations in using the intersection of two machine learning methods to identify hub genes. To address this, we supplemented our analysis by validating the genes selected by each method individually. This includes *SLC16A1* and *ABCG1* identified by LASSO, as well as *C1QB*, *CD5L*, *AXL, DUSP8, CD24, PLG, NRP2*, and *STAB2* selected by SVM-RFE. The results of this analysis are presented in **Supplementary Figure S1** of the **Supplementary Data**. Notably, supplementary analysis revealed significant differences in *CD5L, DUSP8, CD24*, and *SLC16A1* expression between MASH and control samples, with ROC analysis confirming their strong predictive potential.

### Validation in animal experiments

**Figure 9:**
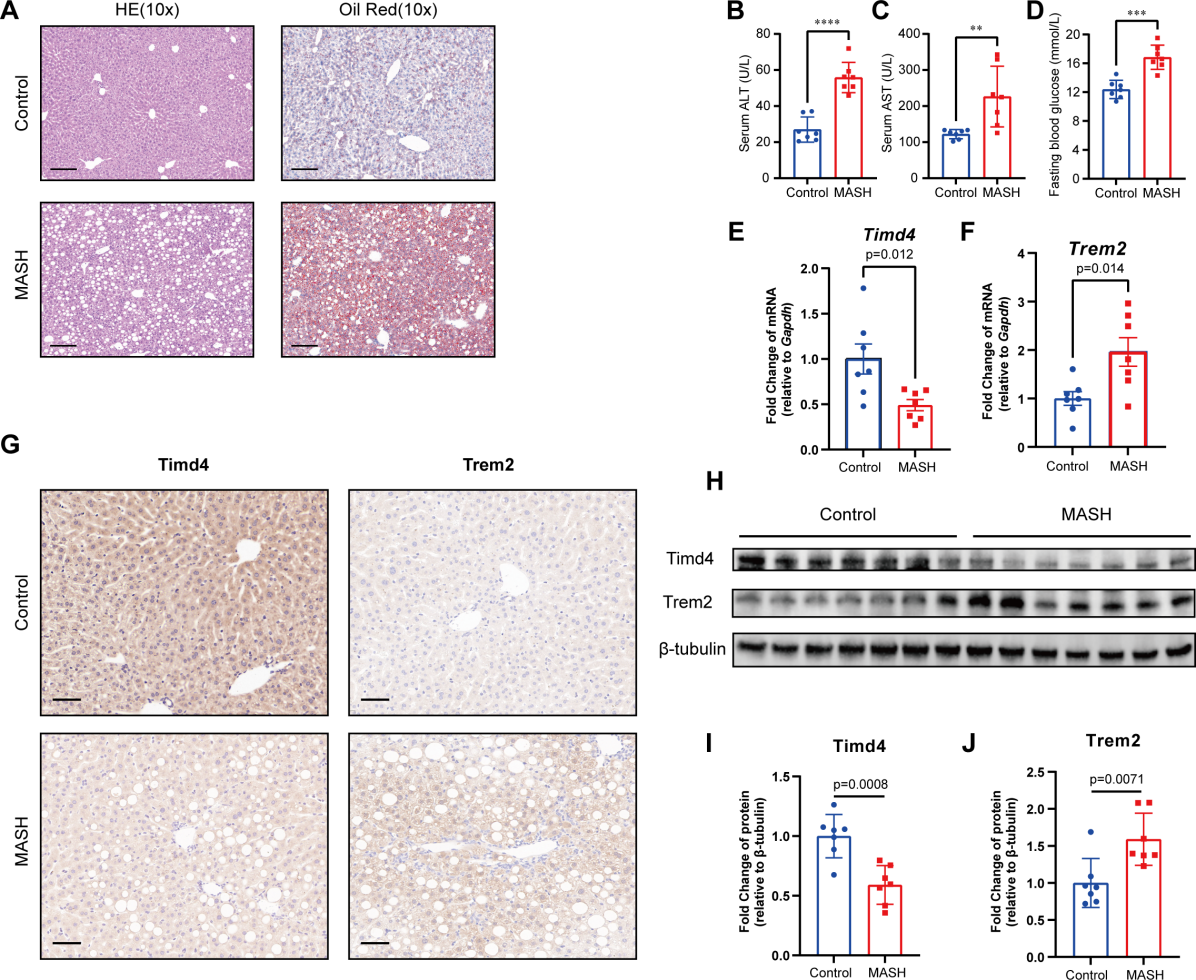
Construction of MASH mice model and validation of hub genes. **(A)** The HE and Oil Red staining in Control and MASH mice. Scale bar, 100 μm. **(B, C)** serum alanine aminotransferase (ALT) and aspartate aminotransferase (AST) level mice model. **(D)** Blood glucose levels after 6 h of fasting. **(E, F)** Relative mRNA levels of *Trem2* and *Timd4* in MASH and Control mice, standardized by *Gapdh*. **(G)** Immunohistochemical staining of TREM2 and TIMD4 in MASH and Control mice. **(H-J)** Relative protein level of TREM2 and TIMD4 in MASH and Control mice, standardized by β-tubulin. ** indicates P<0.01, *** indicates P<0.001, **** indicates P<0.0001.

H&E and Oil Red O staining revealed significant lipid accumulation in the liver tissues of the MASH group, marked by the presence of numerous fat droplets (**Figure 9A**). The measurements of ALT, AST and fasting blood glucose levels showed a notable elevation in the MASH group compared to the control group (**Figures 9B–D)**. Furthermore, the body weight, OGTT, and ITT results indicated that the MASH group had higher values than the control group (**Supplementary Figures S2A–E**). Collectively, these findings suggest the successful establishment of the MASH model.

To explore the role of efferocytosis-related genes in MASH, we evaluated the mRNA and protein levels of key hub genes using RT-qPCR, western blotting, and IHC. The analysis revealed a significant alteration in the expression of *Trem2* and *Timd4* in the MASH group compared to the control group (**Figures 9E–J**). Conversely, no significant differences were observed in *C1qc, Stab1*, and *Dynlt1* (**Supplementary Figures S2F–H**). These results underscore the critical role of *TREM2* and *TIMD4* in MASH development, reinforcing their regulatory impact on disease progression.

## Discussion

Given the escalating prevalence of obesity and metabolic syndrome, MASLD has emerged as a prevalent chronic liver condition globally (36). The onset of MASH, coupled with the surge in patients progressing to cirrhosis and end-stage liver disease necessitating liver transplants, has imposed a substantial financial strain on society (5). Thus, there is a pressing need to discover novel and reliable biomarkers for the diagnosis and treatment of MASH. Efferocytosis, the physiological process whereby phagocytes engulf and eliminate apoptotic cells, has garnered significant research attention due to its role in cardiovascular diseases, ischemic stroke, and cancer (10, 12, 37, 38). However, the mechanisms underlying efferocytosis in MASH pathogenesis and its therapeutic strategies as targets remain largely unexplored. Therefore, our study endeavored to investigate the diagnostic and prognostic values of EFRGs in MASH pathogenesis, identify potential hub genes, and explore latent regulatory targets.

In this study, we thoroughly investigated the differential expression patterns of EFRGs between MASH and control liver samples from the GEO database. Our analysis identified five EFRGs—*TREM2, TIMD4, STAB1, C1QC*, and *DYNLT1*—as being significantly associated with MASH. The distinct differences in the expression of these genes between MASH patients and healthy controls suggested an important role for EFRGs in MASH development and progression. However, upon validating these findings with external datasets and animal models, only *TREM2* and *TIMD4* showed significant differences. This indicated the limitations of relying solely on the intersection of two machine learning methods. To address this, we conducted a supplementary analysis on genes identified by each machine learning method individually. The results revealed that *CD5L, DUSP8, CD24*, and *SLC16A1* were significantly different between MASH and control samples and demonstrated strong predictive power for MASH occurrence (**Supplementary Figure S1**).

The BP analysis of GO enrichment demonstrated that efferocytosis is impaired in MASH, as pathways related to phagocytosis, cholesterol efflux, and apoptotic cell clearance are downregulated (**Figure 3A**) (43). This impairment likely led to the accumulation of apoptotic cells and lipids in the liver, exacerbating inflammation and fibrosis—key features of MASH (44). Besides, the upregulation of pathways involved in the positive regulation of cytokine production indicated a pro-inflammatory environment, consistent with the chronic inflammation observed in MASH patients (**Figure 3A**) (45). The MF analysis further supported this (**Figure 3B**), revealing reduced activities in low-density lipoprotein particle receptor binding, apolipoprotein binding, and scavenger receptor activity, all crucial for maintaining lipid homeostasis. Notably, the upregulation of S100 protein binding, often linked to inflammation and immune responses, underscored the ongoing inflammatory processes in MASH (46). DO enrichment analysis connected the differentially expressed genes to diseases like atherosclerosis (**Figure 3C**), which are associated with metabolic dysfunction and chronic inflammation (47). This suggested that impaired efferocytosis in MASH may also contribute to systemic inflammatory diseases. Supporting this, numerous cohort studies have shown that MASH patients face a significantly increased risk of cardiovascular morbidity and mortality (48, 49). The KEGG pathway enrichment analysis highlighted the significant involvement of pathways related to efferocytosis, complement and coagulation cascades, neutrophil extracellular trap formation, osteoclast differentiation, and cholesterol metabolism (**Figures 3D, E**). These pathways are crucial in local and systemic inflammation, tissue remodeling, and lipid metabolism, emphasizing the multifaceted role of EFRGs in MASH progression. Specifically, our findings for the complement and coagulation cascades pathway are supported by both ssGSEA and PPI analysis, aligning with Sander S.’s research (50), which linked an activated complement system to MASLD. However, the exact mechanisms remain unclear. Molecules involved in efferocytosis, such as *TIMD4* and *C1QC*, may contribute to this process, but further investigation is needed.

In medical research, machine learning is increasingly utilized for accurate diagnosis, prognosis, and treatment forecasting (51, 52). In our study, we employed two machine learning models—LASSO and SVM-REE—to identify genes crucial for MASH diagnosis, focusing on the expression patterns of 39 differentially expressed EFRGs. By intersecting the results of both models, we identified five hub genes: *TREM2, TIMD4, C1QC, STAB1*, and *DYNLT1* (**Figure 4**). However, in subsequent validations using external datasets and animal experiments, only *TREM2* and *TIMD4* were consistently validated, pointing to potential limitations in using the intersection of two machine learning methods to identify core genes. To address this, we separately validated genes identified by each model individually. Beyond *TREM2* and *TIMD4*, *CD5L, DUSP8, CD24*, and *SLC16A1* also showed significant differential expression in MASH. Additionally, ROC analysis showed AUC values above 0.85 for all six genes, indicating strong predictive value for MASH (**Figure 8**, **Supplementary Figure S1**). Therefore, the strategic application of machine learning algorithms can significantly aid in identifying key targets for disease intervention.

TREM2 is an activating receptor highly expressed in tissue macrophages, responsible for detecting apoptotic cells by recognizing exposed phospholipids (39). Previous studies have shown elevated *TREM2* expression in the livers of MASH patients, along with increased circulating levels of soluble TREM2 (53, 54). Our findings further confirmed this at both the transcriptional and protein levels (**Figures 9F–H, J**). Additionally, our GSEA results revealed *TREM2*’s involvement in pathways related to the proteasome, carbohydrate digestion and absorption, and particularly amino acid metabolism (**Figure 5A**). While *TREM2*’s role in glucose and lipid metabolism and its impact on disease onset and progression are well-documented (40, 55), its connection to amino acid metabolism remains unclear. Given the interplay between different metabolic pathways, we hypothesize that *TREM2* may also influence amino acid metabolism. Significantly, Y Eugene Chen’s study reported that a synthesized tripeptide, DT-109 (Gly-Gly-Leu), alleviated MASH in primates, highlighting a potential link between amino acid metabolism and MASH (56). Considering our findings, further research into *TREM2*’s role in amino acid metabolism and its association with MASH progression is warranted. TIMD4 is a phosphatidylserine receptor with a selective expression pattern on antigen-presenting cells, suggesting its involvement in immune-mediated disorders. Research has shown that *TIMD4* knockout mice exhibit exacerbated liver inflammation and hepatic steatosis compared to wild-type counterparts, although the exact mechanism remains unclear. In our study, *TIMD4* expression was found to be reduced in MASH liver samples compared to control liver tissues, at both the transcriptional and protein levels. Additionally, our ssGSEA results suggested that *TIMD4* is involved in regulating oxidative phosphorylation, as well as complement and coagulation cascades (**Figure 5B**). This finding is somewhat consistent with the study by Weiping Zou et al. (57), who revealed that *TIMD4*+ tumor-associated macrophages (TAMs) exhibit high oxidative phosphorylation. Oxidative phosphorylation is often associated with anti-inflammatory metabolic pathways (41). Previous studies have also shown that *TIMD4* can inhibit the activation of NLRP3 inflammasomes and the release of IL-1β, demonstrating anti-inflammatory effects (58, 59). Based on our research, we speculated that the downregulation of *TIMD4* in patients with MASH may lead to increased inflammation due to disturbances in oxidative phosphorylation. Furthermore, the role of *TIMD4* and the complement pathway in MASH warrants further investigation.

MASH is a complex disease with a multifactorial etiology, and recent studies suggest that the immune system plays a pivotal role in its pathogenesis (42). In our study, we employed the CIBERSORT algorithm and scRNA-Seq technology to analyze immune cell infiltration in MASH. We observed an increased proportion of CD4 memory T cells, dendritic cells, and M1 macrophages in MASH liver samples compared to controls (**Figure 7A**). In contrast, the proportions of resting NK cells and M2 macrophages were lower in MASH compared to controls. Macrophages, a heterogeneous population encompassing resting M0, classically activated M1, and alternatively activated M2 phenotypes, are known to accumulate in the liver during MASH (60). The increase in pro-inflammatory M1 macrophages and decrease in anti-inflammatory M2 macrophages aligned with the chronic inflammatory status reported in MASH (45). Our results also revealed a strong correlation between *TREM2* and various immune cell types, such as macrophages and dendritic cells (**Figure 7B**), consistent with previous studies (39). These findings underscored the critical role of the immune system in MASH development and suggest that *TREM2* plays a pivotal role in shaping the immune microenvironment in MASH patients. Additionally, we found significant correlations between *TIMD4* and *C1QC* with M2 macrophages, which are involved in anti-inflammatory responses (61). This suggests that *TIMD4* and *C1QC* may be crucial in resolving inflammation in MASH. Moreover, our scRNA-Seq analysis revealed distinct expression profiles of five hub genes within specific immune cell subsets, such as Kupffer cells, NK cells, and neutrophils (**Figures 7C, D**). These findings suggested that these genes may play critical regulatory roles in hepatic pathological pathways, particularly in orchestrating inflammation and liver fibrosis through the modulation of efferocytosis.

Since we have identified the hub efferocytosis-related genes in MASH, it is essential to pinpoint regulatory factors that modulate these genes, offering direction for future research. To achieve this, we constructed a TF-miRNA-mRNA regulatory network, which highlighted MYC as a key transcription factor and miR-34a-5p and miR-27a-3p as important regulators of the hub genes. MYC is an oncogenic transcription factor, overexpressed in many malignancies and linked to aggressive tumor progression and poor survival outcomes (62). Recent studies, however, propose that MYC functions as a global gene expression amplifier (63). Research has shown that MYC promotes efferocytosis. Xiancai Zhong et al. demonstrated that MYC enhanced efferocytosis and promoted inflammation resolution by driving macrophage M2 polarization (64). Additionally, Ira Tabas and colleagues introduced the concept of efferocytosis-induced macrophage proliferation (EIMP), where MYC plays a central role (65, 66). MYC has also been identified as a promising therapeutic target for MASLD (67, 68). Therefore, further investigation into how MYC regulates efferocytosis through the modulation of hub genes and its impact on MASH is warranted. miRNAs are small, non-coding RNAs that function as post-transcriptional regulators of protein-encoding genes (69). The two miRNAs we identified, miR-34a-5p and miR-27a-3p, have also been recognized as promising targets for the prevention and treatment of MASLD (70, 71). These miRNAs are integral components of the complex regulatory network involving TF-miRNA-mRNA interactions, highlighting the intricate molecular interplay underlying MASH pathogenesis. Understanding this regulatory network could offer valuable insights for therapeutic interventions.

While our study provides valuable insights, it is imperative to acknowledge its limitations. The disparity between RNA-Seq and MASH mice model results implies the complex mechanism of efferocytosis in MASH. Besides, the current work lacks direct cellular evidence, necessitating functional experiments to validate the of these genes in MASH progression. Given the time constraints of this study, we were unable to fully explore the regulatory mechanisms of efferocytosis in MASH. Therefore, future research endeavors focused on these central genes are urgently required to advance our understanding of this complex disease.

## Conclusion

Through a comprehensive bioinformatics approach, our study uncovered key EFRGs associated with MASH. Employing machine learning model, we further identified five significant EFRGs: *TREM2, TIMD4, STAB1, C1QC*, and *DYNLT1*. Our results revealed that *TREM2* exhibited elevated expression in MASH patients, whereas *TIMD4* demonstrated reduced expression. Furthermore, diagnostic models leveraging either *TREM2* or *TIMD4* as biomarkers made remarkable diagnostic accuracy in NASH, underscoring their potential clinical utility. In conclusion, our findings offer new opportunities and promising therapeutic targets for MASH efferocytosis research.

## Data Availability

The datasets presented in this study can be found in online repositories. The names of the repository/repositories and accession number(s) can be found in the article/**Supplementary Material**.
