# Supplementary material for: Identification and validation of efferocytosis-related biomarkers for the diagnosis of metabolic dysfunction-associated steatohepatitis based on bioinformatics analysis and machine learning
Source: Front Immunol. 2024 Oct 21;15:1460431. doi: 10.3389/fimmu.2024.1460431 (PMC11532026; doi:10.3389/fimmu.2024.1460431)
Supplement: Supplementary file 2 [file DataSheet2.docx]

**SUPPLEMENTAL DATA**

**Identification and validation of efferocytosis-related biomarkers for the diagnosis of metabolic dysfunction-associated steatohepatitis based on bioinformatics analysis and machine learning**

Chenghui Cao^1,2^, Wenwu Liu^1^, Xin Guo^1^, Shuwei Weng^1^, Yang Chen^1^, Yonghong Luo^1^, Shuai Wang^1^, Botao Zhu^1,2^, Yuxuan Liu^1,2^, Daoquan Peng^1*^

1. Department of Cardiology, Research Institute of Blood Lipids and Atherosclerosis, The Second Xiangya Hospital, Central South University, Changsha, Hunan 410011, China.
2. Xiangya School of Medicine, Central South University, Changsha, China.

* Corresponding author:

Daoquan Peng

Department of Cardiology, Research Institute of Blood Lipids and Atherosclerosis, The Second Xiangya Hospital, Central South University, Changsha, Hunan 410011, China.

E-mail: pengdq@csu.edu.cn

**Supplemental Figures**
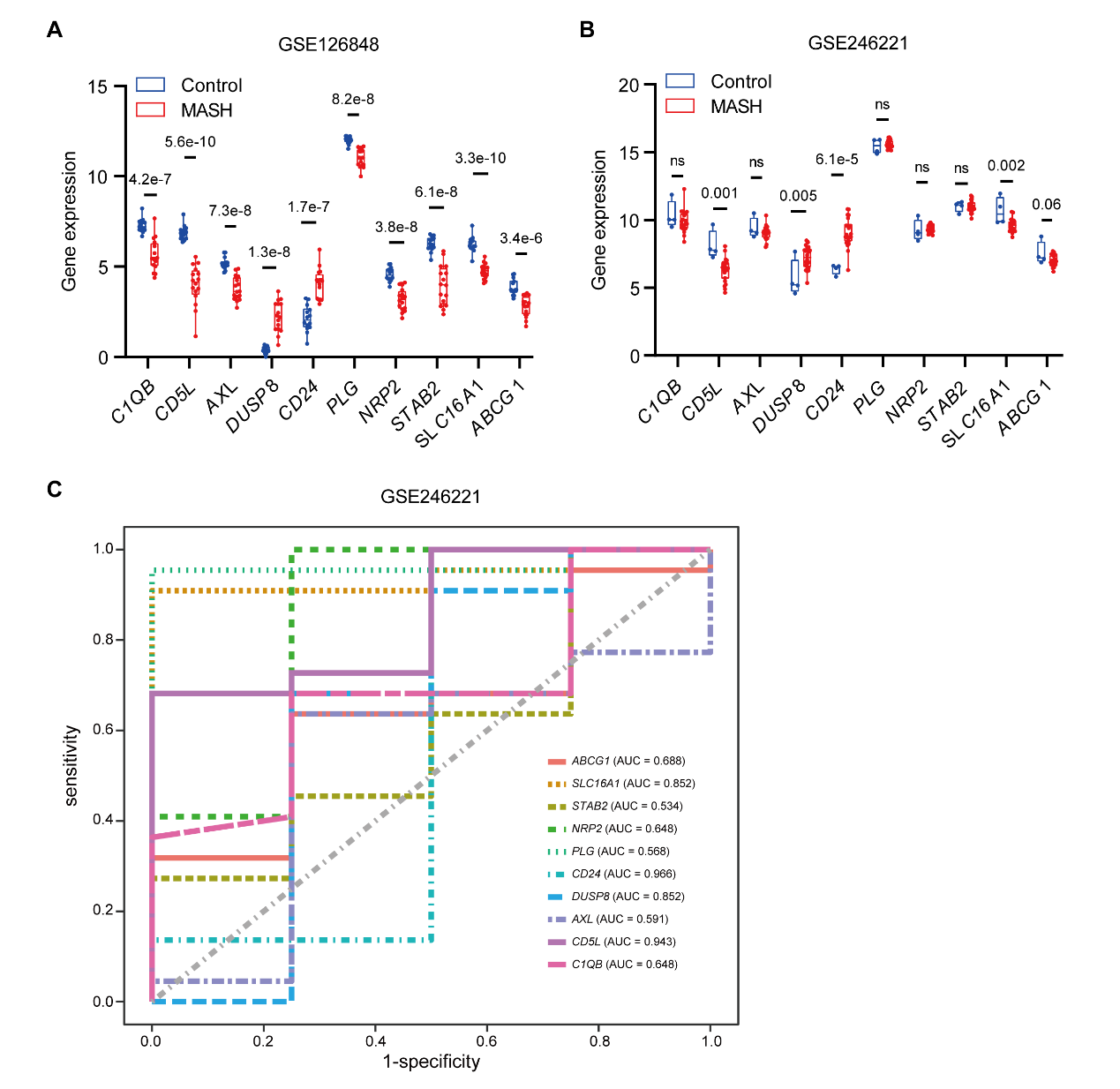


**Figure S1. Validation of genes screened by LASSO and SVM-RFE model.** (**A**) Boxplots depicting the ten individually screened genes using the LASSO or SVM-RFE method between MASH and control samples in the GSE126848 dataset. (**B**) Boxplots depicting the ten individually screened genes using the LASSO or SVM-RFE method between MASH and control samples in the GSE246221 dataset. (**C**) The ROC results of ten individually screened genes.


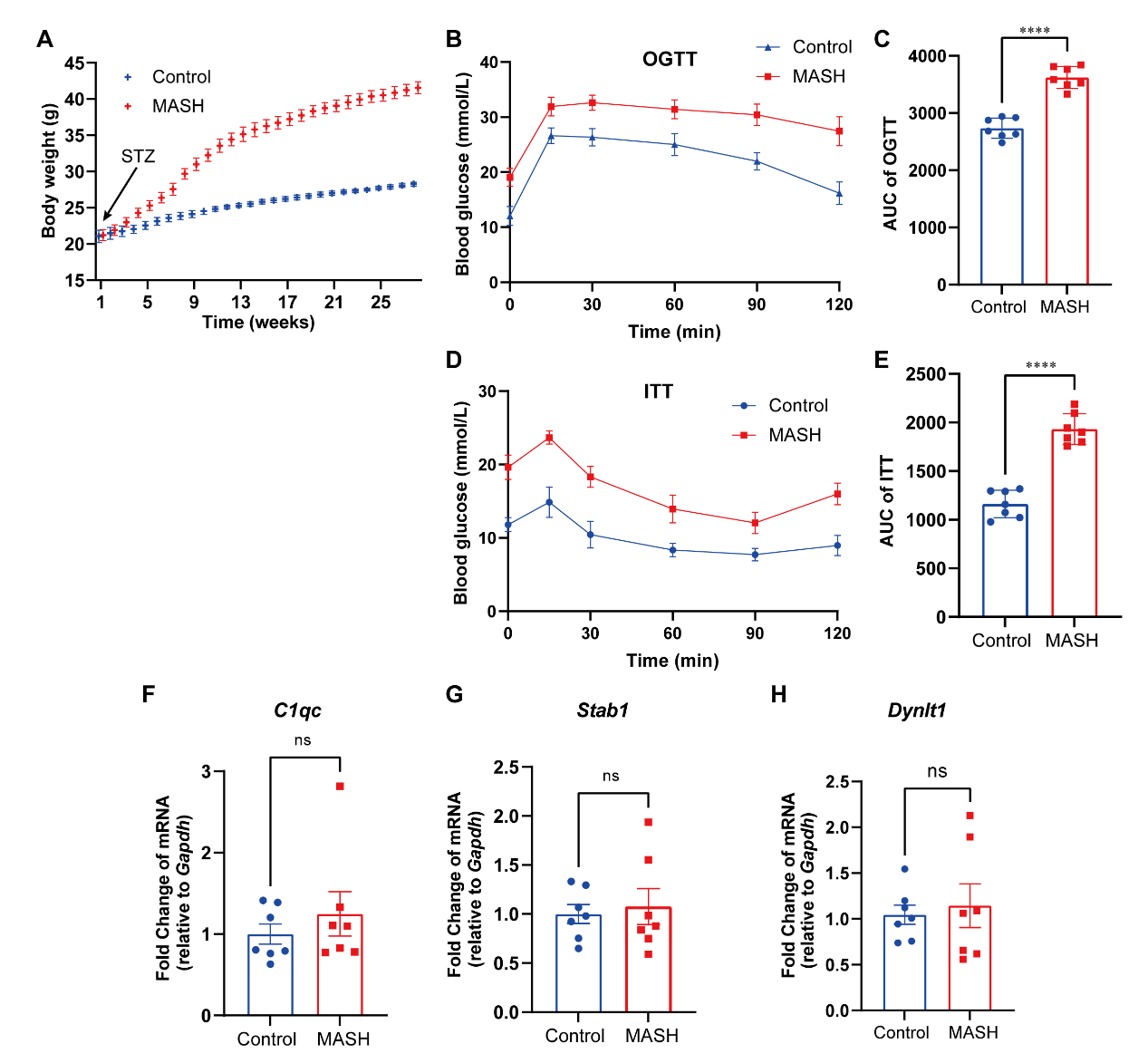


**Figure S2. Construction of MASH mice model and validation of hub genes.** (**A**) Body weight trends. (**B-C**) Oral glucose tolerance test (OGTT) after overnight of fasting at 20 weeks of age. (**D-E**) Insulin tolerance test (ITT) after 4h of fasting at 20 weeks of age. (**F-H**) Relative mRNA levels of *C1qc*, *Stab1* and *Dynlt1* in MASH and Control mice, standardized by *Gapdh*. ns indicates not significant, **** indicates P<0.0001.

**Supplementary Table S2. Primer sequences used for RT-qPCR**

| Gene symbol | Primer sequences |
| --- | --- |
| *Gapdh* Fw (5’-3’) | AAGGTCATCCCAGAGCTGAA |
| *Gapdh* Rv (5’-3’) | AGGAGACAACCTGGTCCTCA |
| *Trem2* Fw (5’-3’) | CTGGAACCGTCACCATCACTC |
| *Trem2* Rv (5’-3’) | CGAAACTCGATGACTCCTCGG |
| *Timd4* Fw (5’-3’) | AGCTTCTCCGTACAGATGGAA |
| *Timd4* Rv (5’-3’) | CCCACTGTCACCTCGATTGG |
| *Stab1* Fw (5’-3’) | GGCAGACGGTACGGTCTAAAC |
| *Stab1* Rv (5’-3’) | AGCGGCAGTCCAGAAGTATCT |
| *C1qc* Fw (5’-3’) | CCCAGTTGCCAGCCTCAAT |
| *C1qc* Rv (5’-3’) | GGAGTCCATCATGCCCGTC |
| *Dynlt1* Fw (5’-3’) | GACAGCTCCACAGACGGAAG |
| *Dynlt1* Rv (5’-3’) | GCAAGGTGGTCAGATGGACA |
